# The Potential of Non‐invasive Neurostimulation, tDCS for Cognitive Decline in Alzheimer’s Disease: Through the Lens of an Ongoing Clinical Trial

**DOI:** 10.1002/alz.087482

**Published:** 2025-01-03

**Authors:** Jordan Vanzyl, Ivan Zhen, Kevin Ha, Itzel Flores, Melany Taranto, Mairim Melecio‐Vazquez, Emmeline Ayers, Erica Weiss, Jessica L Zwerling, Helena M Blumen, Joe Verghese, Helena Knotkova

**Affiliations:** ^1^ MJHS Institute for Innovation in Palliative Care, New York, NY USA; ^2^ Albert Einstein College of Medicine, Bronx, NY USA; ^3^ Montefiore Einstein, Bronx, NY USA; ^4^ Albert Einstein College of Medicine, New York, NY USA

## Abstract

**Background:**

Transcranial direct current stimulation (tDCS) is a non‐invasive neuromodulation method. Short‐term tDCS protocols have shown positive effects on cognitive outcomes in Alzheimer’s Disease (AD) populations^1‐5^. Less is known about the long‐term benefits of tDCS on cognition in AD. Here, we discuss the evidence on efficacy of tDCS in AD and the potential of this novel technique applied in home settings^6‐8^ through the lens of our ongoing long‐term, large‐scale (N = 100) randomized controlled clinical trial in patients with mild‐to‐moderate AD. This RCT aims to determine the effects of tDCS on cognition, mood, quality of life and functional/structural brain connectivity, and to explore the durability of these effects after 9‐months.

**Method:**

Participants age ≥60 years with mild‐to‐moderate stage AD and the ability to provide informed consent are eligible. Participants receiving/have received monoclonal antibody treatment for AD or have history or head trauma or seizures are excluded. Participants are randomized to receive active tDCS at 2mA or sham delivered with two saline‐soaked electrodes placed over the dorsolateral prefrontal cortex for 30 minutes/day, 5 days/week for 6‐months at home.

**Result:**

The study protocol has been developed and approved by the IRB. It includes 6 study visits (V1‐6) over 9 months. Eligible participants undergo in‐person tDCS training (V2), followed by a refresh training and the first tDCS session under supervision of study personnel (V3). Daily tDCS sessions proceed at home for 6 months, with a daily remote supervision via phone/video. Outcome assessments post‐intervention occur at 6, 7 and 9‐months (V4‐6). Our team developed and implemented an enhanced remote monitoring procedure (ERMP) that promotes safety and protocol adherence by monitoring skin condition and allowing for quality checks of electrode placement. The study protocol has been successfully implemented and is ongoing. To date, 101 participants have been consented/screened; of those, 62 were eligible and randomized to apply tDCS at‐home for 6‐months. Enrollment will continue until 2025.

**Conclusion:**

There is preliminary evidence on the potential of tDCS for symptom management in AD. Our study will expand the understanding of the long‐term benefits of tDCS in AD. If effective, this non‐pharmacological intervention could become an important strategy for symptom management in AD patients.

